# Supplementary material for: Remote Hospital Care for Recovering COVID-19 Patients Using Telemedicine: A Randomised Controlled Trial
Source: J Clin Med. 2021 Dec 17;10(24):5940. doi: 10.3390/jcm10245940 (PMC8706980; doi:10.3390/jcm10245940)
Supplement: Supplementary file 1 [file jcm-10-05940-s001.zip › jcm-1501604-supplementary.docx]

Supplementary Figure S1: Manual Early@home

**Manual Early@home *(translated from Dutch)***

Table of content

1. Introduction
2. Measurement moments
3. Manual Luscii app
4. Manual pulse oximeter
5. Personal treatment plan
6. Who to call?
7. Important notions on logging in and privacy

1. Introduction

You receive this information because you are participating in Early@home. U were randomized to the group of patients who will leave the hospital early. This means you will spend the last days of hospital care at home. During these days you will enter measurements in the Luscii app three times daily. If you do not feel well, you can enter extra measurements. Every day you will be contacted by telephone to discuss the measurements. When you will no longer be in need of hospital care, the measurements will end.

In this manual you will find all information on entering measurements and the use of the pulse oximeter. A personal treatment plan was added so you know what to do with the results. Information on who to call in which situation can be found on the last page.

2. Measurement moments

The telemonitoring consists of 3 measurement moments and 1 contact moment with the medical monitoring center every day:

Between 9.00-10.00: 1^st^ time entering measurements in app incl. temp and saturation 5 min

Between 11.00-12.00: Contact moment with hospital (we will contact you) 10 min

Between 14.00-15.00: 2^nd^ time entering measurements in app incl. temp and saturation 5 min

Between 21.00-22.00: 3^rd^ time entering measurements in app incl. temp and saturation 5 min

The measurements will start as soon as you arrive home. For example, if you arrive home at 16.00, the first measurement will be at 21.00. The following days you will follow the schedule above. You can find the actions of the day in your personal treatment plan.

Do you feel unwell in between measuring moment? Enter an extra set of measurement in the app and follow your personal treatment plan. If you are experiencing severe symptoms you can also contact the hospital directly. In case of emergency call 112.

The hospital will contact you every day to discuss your treatment. If you have recovered enough and no longer need hospital care, we will hand you over to your general practitioner. You do no longer have to enter measurements in the app. You can send the pulse oximeter back to the hospital by mail using the return envelope.

3. Manual Luscii app

You downloaded the Luscii app in the hospital and you have received an account. To enter measurements, please open the app on your phone.

- Select the app ‘Luscii’
- You now see ‘Actions’. These are the questions you have to answer today.
- Start with ‘Temperature’. Push start.
- You can now read the information on how to enter a measurement. Push next.
- Enter your temperature. Push next.
- In the next screen you will see your temperature. Push send.
- Your temperature is now sent. You will see a green check mark next to ‘Temperature’.
- You can now go on to the next question. Again, push start.
- You are done if all questions have a green check mark.
- To be able to enter ‘Saturation’ you will need a pulse oximeter, please see the manual below.

4. Manual pulse oximeter

You received a pulse oximeter from the hospital. Oxygen saturation is a measure to indicate the amount of oxygen in the blood. The pulse oximeter can also measure your heartrate. We will not use this measurement in this case.

- Always measure your saturation at rest. If you just delivered physical effort (e.g. walked up the stairs), take 5 minutes of rest before you measure your saturation.
- Turn the pulse oximeter on by pushing the button on top.
- Place your finger in the pulse oximter with your fingernail facing down.
- Wait for 10 seconds in order to get a reliable measurement. If the number keeps increasing or decreasing, wait until it has stabilized.
- The number on the left, marked by ‘SpO2%’, is the oxygen saturation. This is the number you will have to enter into the Luscii app.
- You can ignore the number on the right, this is the heartrate.
- Take your finger out of the pulse oximeter. The pulse oximeter will shut down by itself.

Figure S1 - Manual Early@home


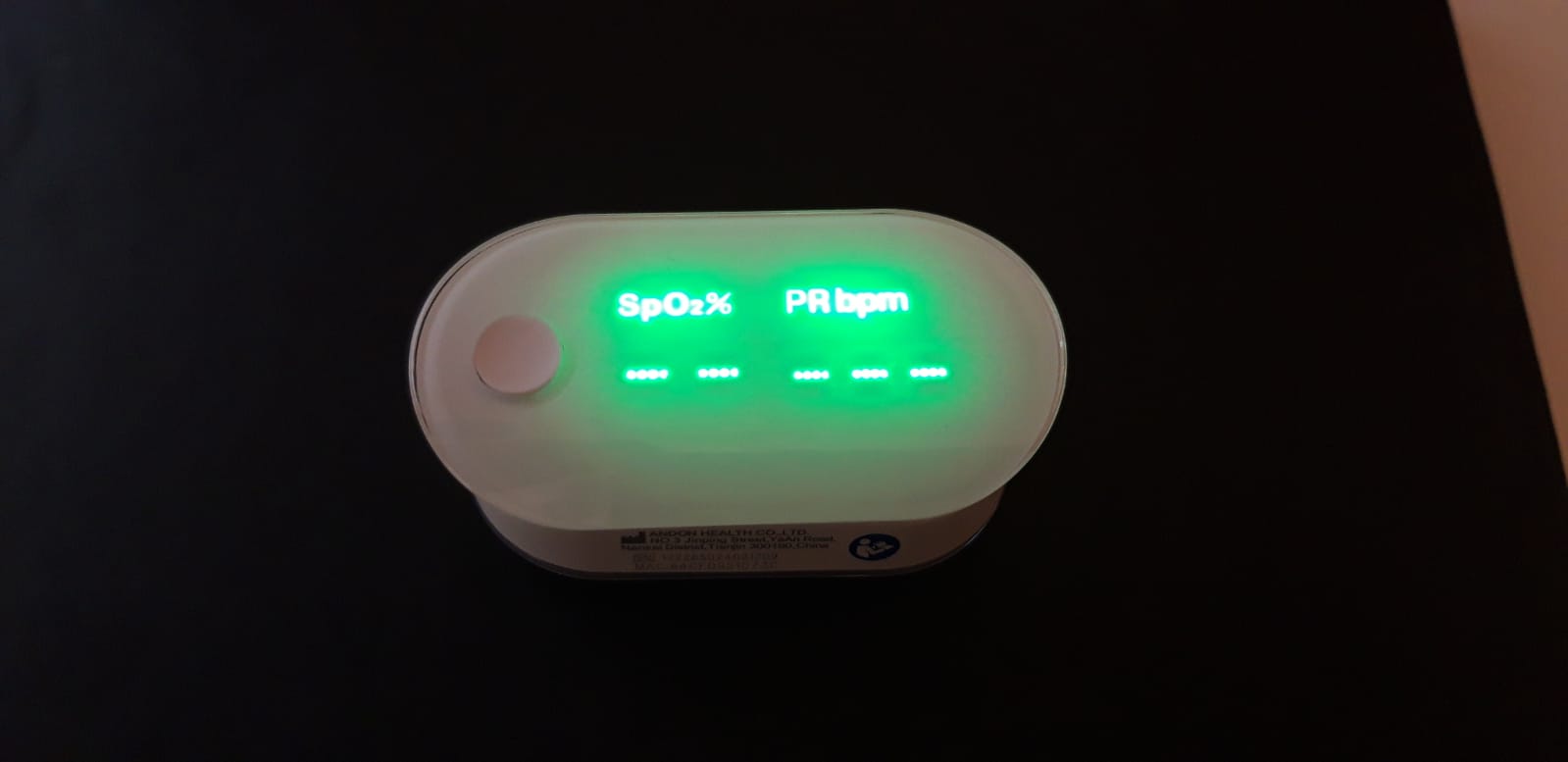


**Oxygen saturation**


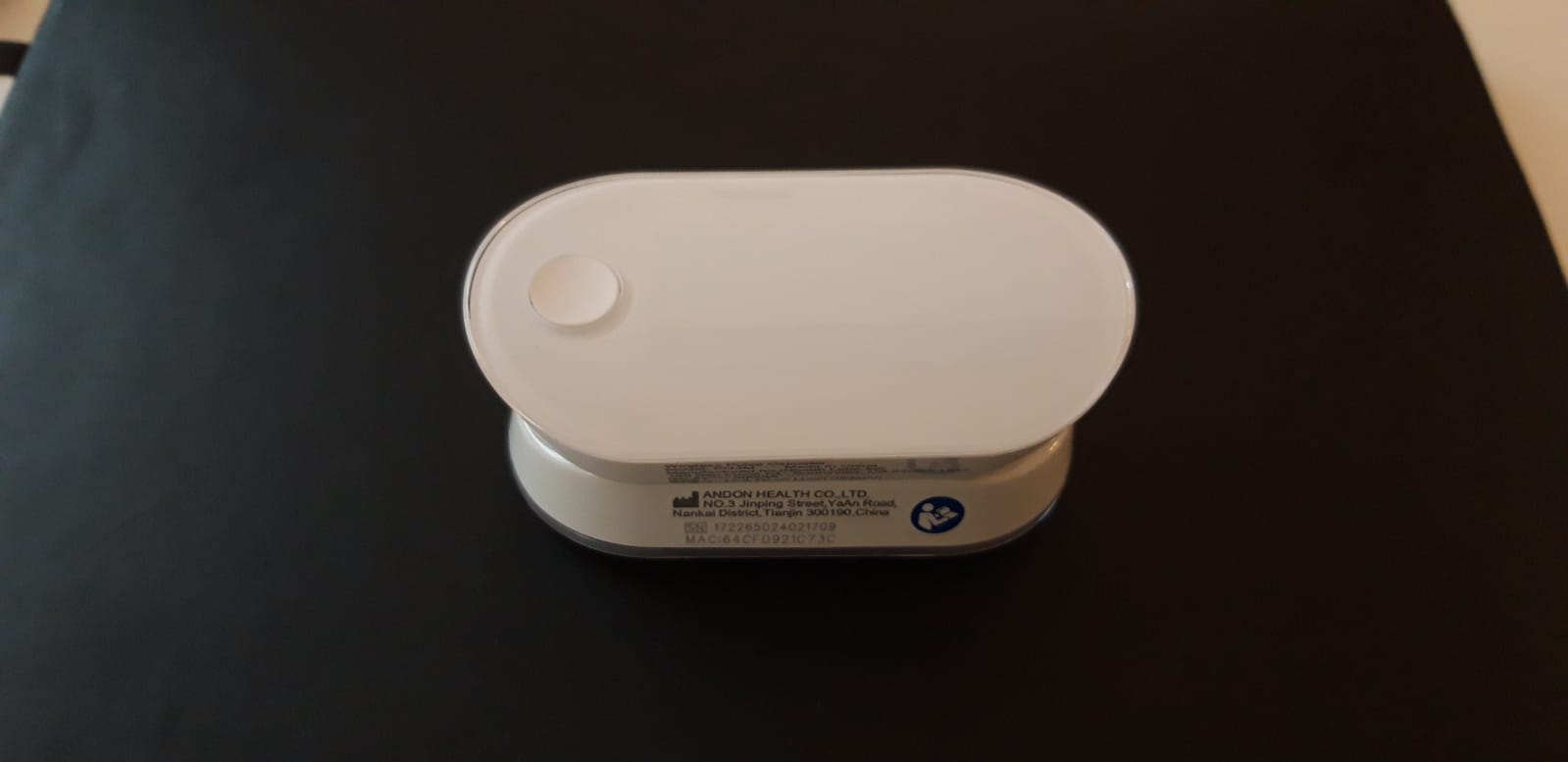


**On button**

Aanknop


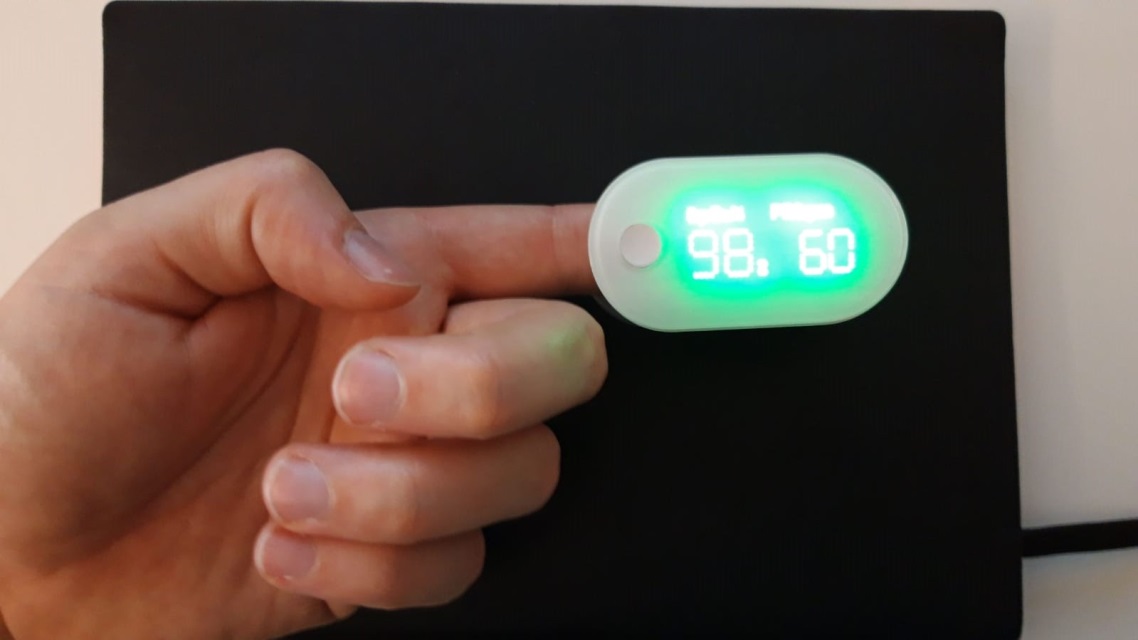


**This is the number**

**you enter in the app**


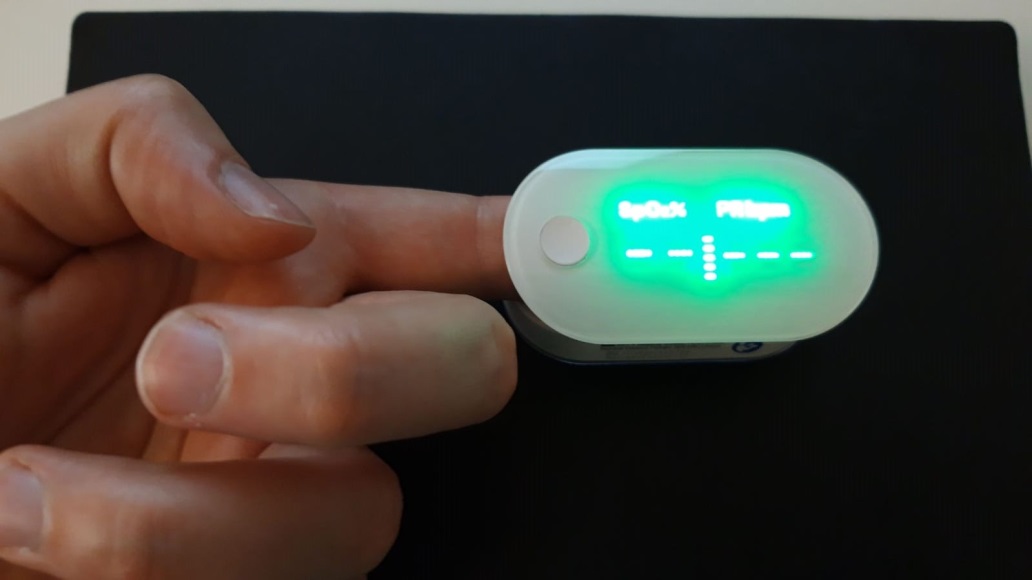


**Place your finger in the**

**pulse oximeter with the**

**nail facing down**

5. Personal treatment plan

Below you find your pesonal treatment plan. The physician will write down the right values on the dotted lines.

Do you feel unwell between two measurement moment? Please enter an extra set of measurements in the app.

- **Your oxygen saturation is ______ or lower** > Take a seat and rest. Measure your saturation again after 5 minutes.
  - Still ______ or lower after 5 minutes? Call us immediately (see ‘Who to call?’)
  - Higher than ______ after 5 minutes? See below.
- **Your oxygen saturation is ______ up to ______.**
  - Do you feel ill, short of breath, or do you have a fever? Call us immediately (see ‘Who to call?’).
  - Do you have minor or no symptoms? You do not have to do anything. You will be contacted by the hospital during our next contact round (11.00 or 15.00).
- **Your oxygen saturation is ______ or higher** > You do not have to do anything

***Alarm symptoms***

Do you experience any of these symptoms? Call us **ALWAYS**  and **IMMEDIATELY:**

- You have trouble breathing
- Your lips are blue
- You are breathing very often even though you are not exercising
- You feel a lot worse all of a sudden

In case you cannot wait for our assistance, please call 112 directly.

Are you admitted to a different hospital than the UMC Utrecht? Please ask your physician to contact us via *<telephone number>.*

6. Who to call?

**> Do you have to call us immediately according to your treatment plan, or do you want to contact the hospital for another reason? Please call:**

Between 09.00-16.00 Medical Monitoring Center, *<telephone number>.*

Between 16.00-09.00 COVID-19 physician on call, *<telephone number>.*

**> Are your symptoms so severe that you can no longer wait? Please call 112 immediately.**

**> Do you have symptoms not related to COVID-19?** Please call your general practitioner.

> In case you have a different question or remark, please use the phone numbers below, depending on your question:

**Questions regarding the study:**

Researcher *<telephone number>.*

Supervisor *<telephone number>.*

**Logistics (eg returing the pulse oximeter):**

Bureau Zorgbemiddeling UMCU (BZU) *<telephone number>.*

**Technical difficulties with app or pulse oximter:**

Helpdesk Luscii *<telephone number>.*

7. Important notions on logging in and privacy

You will enter personal data into the Luscii app. Of course, these data have to be secured. Therefore you log into the app using a password you receive via email. However we recommend you also have a pin code or password for you telephone, to prevent others from entering the app. The data you enter will be stored in the app. Do you want your data to be removed? Please contact the app developer Luscii. The data that stored for research will remain stored anonymously according to research guidelines.


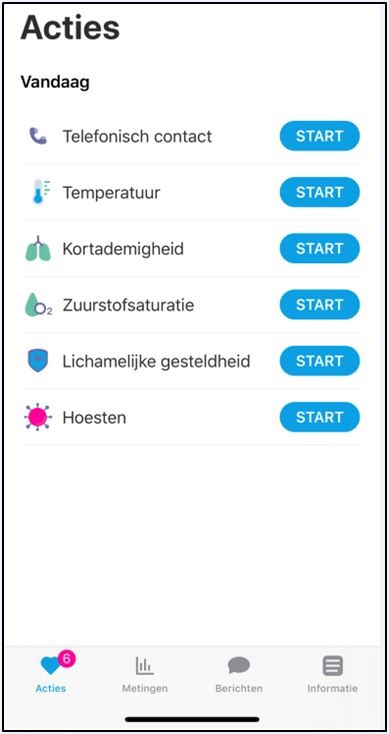


Supplementary Figure S2: App questionnaire

**
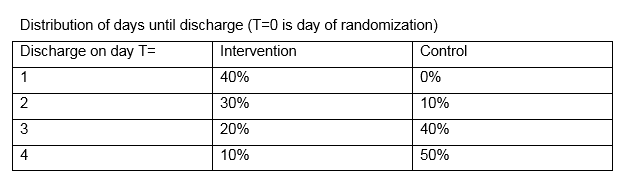
**

Supplementary Table S1: Assumed distribution of days until discharge
